# An evaluation of COVID-19 serological assays informs future diagnostics and exposure assessment

**DOI:** 10.1038/s41467-020-17317-y

**Published:** 2020-07-06

**Authors:** Corine H. GeurtsvanKessel, Nisreen M. A. Okba, Zsofia Igloi, Susanne Bogers, Carmen W. E. Embregts, Brigitta M. Laksono, Lonneke Leijten, Casper Rokx, Bart Rijnders, Janette Rahamat-Langendoen, Johannes P. C. van den Akker, Jeroen J. A. van Kampen, Annemiek A. van der Eijk, Rob S. van Binnendijk, Bart Haagmans, Marion Koopmans

**Affiliations:** 1 Department of Viroscience, Erasmus MC, Rotterdam, The Netherlands; 2 Department of Internal Medicine, Section of Infectious Diseaseas, Erasmus MC, Rotterdam, The Netherlands; 3 Department of Medical Microbiology, Radboud University Medical Centre, Nijmegen, The Netherlands; 4 Department of Intensive Care, Erasmus MC, Rotterdam, The Netherlands; 5 Center for Infectious Disease Control, RIVM, Bilthoven, The Netherlands

**Keywords:** Viral infection, Laboratory techniques and procedures, Medical research

## Abstract

The world is entering a new era of the COVID-19 pandemic in which there is an increasing call for reliable antibody testing. To support decision making on the deployment of serology for either population screening or diagnostics, we present a detailed comparison of serological COVID-19 assays. We show that among the selected assays there is a wide diversity in assay performance in different scenarios and when correlated to virus neutralizing antibodies. The Wantai ELISA detecting total immunoglobulins against the receptor binding domain of SARS CoV-2, has the best overall characteristics to detect functional antibodies in different stages and severity of disease, including the potential to set a cut-off indicating the presence of protective antibodies. The large variety of available serological assays requires proper assay validation before deciding on deployment of assays for specific applications.

## Abstract (web summary)

SARS-CoV-2 is causing a global pandemic in which the implementation of serology can support decision making in different scenarios. Here, the authors compare the outcome of eight commercially available assays to virus neutralization and discuss their use in diagnostics and exposure assessment of SARS-CoV-2.

## Introduction

The novel severe acute respiratory syndrome coronavirus 2 (SARS-CoV-2) was first reported in late 2019 to cause coronavirus disease (COVID-19). The rapid global spread and exponential growth of the pandemic wave have stretched the limits of the available healthcare and intensive care unit capacity. Since the initial notification of an outbreak on December 31st, the global response has transitioned from the initial policy of active case finding and containment to an increasingly complex package of confinement measures including closures of schools, implementation of travel restrictions, and physical distancing measures. At present, given the global circulation of SARS-CoV-2, the consensus is that elimination of the virus is no longer feasible, and that longer-term strategies are needed that strike a balance between the economically and socially damaging (near) lockdown approaches and full release of any control measures. There is wide agreement that, in the latter situation, rapid resurgence would be very likely, with modeled epidemic peaks potentially exceeding the current healthcare capacity^1^.

The so-called exit strategy is defined as the transition from the current approach, which focuses entirely on flattening the peak of the COVID-19 emergence curve, to the transition phase in which restrictions are gradually lifted. The gradual lifting of control measures will require active surveillance to allow early detection of new cases or clusters, coupled with contact tracing and quarantine, most likely combined with continued physical distancing recommendations and enhanced protection of those at-risk from most severe disease. A key knowledge gap is the level and duration of protective immunity in the population at large and in specific groups, including persons with different clinical severity^1,2^.

To assess the extent of virus circulation in the community, and the likelihood of protection against a re-infection, there is a crucial need to add serology to the testing algorithms. The required performance of a serological assay will depend on the specific aim of testing, which may be either population screening (in the general population or at-risk populations) or diagnostic support. We recently showed that antibodies directed against the S1 subunit of the SARS-CoV-2 spike protein and specifically to the receptor binding domain (RBD) within the S1 subunit strongly correlate with virus neutralization^3^. The likelihood of predicting protective antibody responses will thus increase when using either S1 antigens or RBD in the assay. The specificity of serological tools detecting antibodies against SARS CoV-2 might be hampered by the presence of antibodies against other circulating coronaviruses in the population, and thus testing for cross reactivity is crucial. When selecting an appropriate assay for a specific purpose, decision making should include the available knowledge on antibody specificities, kinetics, and functions^4^. The limited knowledge on antibody kinetics in emerging virus infections is always a challenge for design and validation of serological assays during an outbreak. Recent studies in COVID-19 patients have shown that in both hospitalized patients and patients with mild disease, seroconversion rates reach 100% after 10–14 days, and that antibody levels may correlate with clinical severity^2,3,5^. This is in line with observations in Middle East Respiratory Syndrome coronavirus (MERS-CoV) infection, in which antibody responses varied depending on disease severity, with mild and asymptomatic infections resulting in weaker immune responses^6^. Therefore, for meaningful interpretation of serological assays and extrapolation of results to population screening, sufficient samples from persons with mild and asymptomatic disease should be included in validation studies.

In our study we compare three platforms, which are widely used in diagnostic laboratories (three rapid tests, four ELISAs, and a high throughput chemiluminescent assay (CLIA)), which can be used to address different needs: for individualized (home) testing, as supplement to diagnostics and in population screening. We analyze their performance in correlation to an in-house virus neutralization assay^3^, which is currently the gold standard when assessing protective immunity against SARS CoV-2.

## Results

### Patient diagnostics

**Fig. 1:**
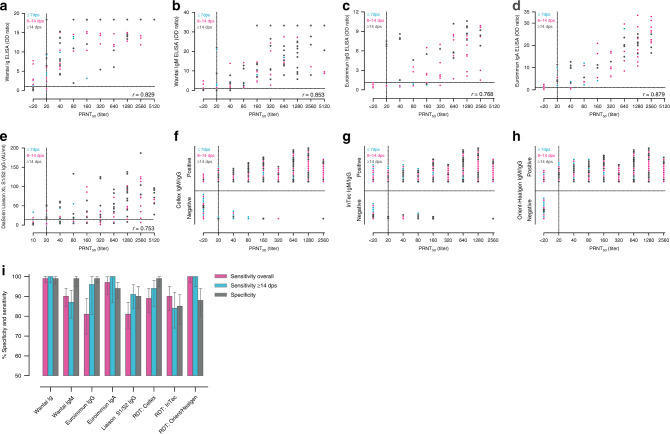
Performance of commercial assays for the detection of SARS-CoV-2-specific antibodies. Correlation of SARS-CoV-2 neutralizing antibody titers tested by a plaque reduction neutralization assay (PRNT50) to antibodies measured by selected assays. (**a**) Wantai Ig total ELISA, (**b**) Wantai IgM ELISA, (**c**) Euroimmun IgG ELISA, (**d**) Euroimmun IgA ELISA, (**e**)  DiaSorin Liaison XL IgG chemiluminescence immunoassay, (**f**) Cellex IgM/IgG, (**g**) InTec IgM/IgG, (**h**) Orient gene/Healgen IgM/IgG. Turquoise dots indicate patient specimen collected ≤7 days post onset of  symptoms  (dps), magenta  dots indicate samples collected from 8–14 dps, gray dots indicate specimen collected more than 14 dps. Dotted lines indicate the cut-off for positivity of each assay, as indicated by the manufacturer: Wantai ELISAs, OD ratio > 1; Euroimmun ELISAs, OD ratio > 1.1; DiaSorin Liaison IgG >15 AU/ml. OD: optical density, AU: arbitrary units, r: correlation coefficient. (**i**) Percentages of specificities and sensitivities of the various platforms tested. The arrow bar indicates the upper and lower limit of the 95% CI. Source data are provided as a Source Data file.

Serological testing to support clinical diagnostic work-up is mostly requested in hospitalized patients. This can be for example when SARS CoV-2 RNA diagnostic testing remains negative in a patient despite a strong clinical suspicion or for patients whose samples during the symptomatic phase were not collected. Other patients in whom antibody testing can be very valuable are those who have been hospitalized for weeks and in whom a PCR test continues to be positive with increasing cycle threshold values. In these patients, the detection of virus neutralizing antibodies can help with the decision to stop using personal protective equipment. In these patients usually the serological results are interpreted by laboratory staff and there is a possibility to test follow-up sera or perform confirmation serological testing. When comparing the laboratory assays in patients with different severity and stages of disease (overall), and in patients tested more than 14 days post-onset of disease, the RBD antigen based Wantai total Ig assay performed best (Fig. 1, Table 1). Both IgG assays targeting the S antigen (Euroimmun and Liaison) lacked sensitivity in hospitalized patients as the sample set included early time-points. The S1 based IgA assay by Euroimmun, in contrast, had a good sensitivity, and showed the best quantitative relationship, specifically once neutralizing titers were higher than 80 (PRNT50 units), upon which the RBD Ig assay becomes non-linear. IgA testing will detect both early and memory IgA responses and will thus be a useful addition to IgG assays. The possible relevance of quantitative antibody measurements will need to be assessed when results of longer-term patient follow-up studies become available. An alternative of the PRNT50 can be the use of a surrogate virus neutralization test for SARS CoV-2 (as recently produced by Genscript, USA) which allows direct quantification. Extensive studies on the performance have however not yet been published.

**Table 1:** A summary of the performance characteristics of eight commercial COVID-19 serology assays.

Assay		Wantai Ig	Wantai IgM	Euroimmun IgG	Euroimmun IgA	Liaison	Cellex	Intec	Orient/Healgen
Platform		ELISA	ELISA	ELISA	ELISA	CLIA	RDT	RDT	RDT
Antigen		RBD	RBD	S1	S1	S1 and S2	S and N	N	S and N
Specificity									
	n/N	145/146	144/146	156/157	147/157	119/132	97/98	83/98	87/98
	Value	0.99	0.99	0.99	0.94	0.90	0.99	0.85	0.88
	95% CI	0.96–1.0	0.951–1.0	0.97–1.0	0.89–0.97	0.84–0.95	0.95–1.0	0.76–0.91	0.80–0.94
Sensitivity									
Overall	n/N	186/187	167/185	61/75	73/75	134/165	101/113	102/113	113/113
	Value	0.99	0.90	0.81	0.97	0.81	0.89	0.90	1.0
	95% CI	0.97–1.0	0.85–0.94	0.71–0.89	0.91–1.0	0.74–0.87	0.82–0.94	0.83–0.95	0.97–1.0
>14 dps	n/N	117/117	100/115	26/27	27/27	95/104	63/67	55/65	67/67
	Value	1.0	0.87	0.96	1.0	0.91	0.94	0.84	1.0
	95% CI	0.97–1.0	0.79–0.93	0.81–1.0	0.87–1.0	0.84–0.96	0.85–0.98	0.74–0.92	0.95–1.0
Mild	n/N	64/64	54/62	1/3	3/3	46/55	33/37	26/36	37/37
	Value	1.0	0.87	0.33	1.0	0.84	0.89	0.72	1.0
	95% CI	0.94–1.0	0.76–0.94	0.0084–0.91	0.29–1.0	0.71–0.92	0.75–0.97	0.55–0.86	0.91–1.0
Overall PPV									
Population prevalence	4%	0.86	0.73	0.84	0.39	0.26	0.78	0.20	0.26
	50%	0.99	0.99	0.99	0.94	0.89	0.99	0.86	0.89
	95%	1.0	1.0	1.0	1.0	0.99	1.0	0.99	0.99
Overall NPV									
Population prevalence	4%	1.0	1.0	0.99	1.0	0.99	1.0	1.0	1.0
	50%	0.99	0.91	0.84	0.97	0.83	0.9	0.9	1.0
	95%	0.91	0.35	0.22	0.65	0.20	0.33	0.31	1.0

Sensitivity of the assays was determined by comparing the outcome to virus neutralization (PRNT> 20). Patients were considered mild if they have not been admitted to a hospital. The PPV was calculated for 3 scenarios: 4 % seroprevalence in a general population. 50% seroprevalence in a high risk sub-population and 90% seroprevalence in hospitalized patients suspect for COVID-19. Ig: immunoglobulin, ELISA: enzyme linked immunosorbent assay, CLIA: chemiluminescence immune assay, RDT: rapid diagnostic test, n:number of positives, N: total number tested, CI: confidence interval, dps: days post onset of symptoms, PPV: positive predictive value, NPV: negative predictive value, PRNT: plaque reduction neutralization test.

Despite the differences in sensitivity, all laboratory assays had sufficient positive predictive value (PPV) in COVID-19 hospitalized patients when assuming an expected seroprevalence in this population of ≥50% (Table 1), or when using serology as an adjunct to RT-PCR testing to monitor the clinical course of illness. Their application as sole diagnostic, however—for instance in primary care, where the seroprevalence will be much lower—will be more challenging as illustrated by the variation in PPV of the assays with a seroprevalence estimate of 4%, which is the level currently observed in the Netherlands^7^.

In addition to specialized ELISA assays used in laboratory settings, a wide range of rapid diagnostic tests (RDT) has been put on the market, triggering the question whether they can be used in patient care or for triage in a medical care facility. We selected three RDTs by following criteria (1) preferably targetting the spike of SARS-CoV-2 (2) at least European Conformity (CE) marking or other authorization, and (3) sufficient production capacity. The RDTs provide qualitative (yes/no) results, which does not allow quantification or the definition of a cut-off for neutralization (Fig. 1f–h). All three RDTs had a sufficient PPV in high seroprevalence scenarios, which implies that there might be a role for the RDTs when used for the individual patient with a sufficiently high pretest probability as an add on to PCR based diagnostics. The high negative predictive value (NPV) of the RDTs in a low seroprevalence scenario could offer opportunities for the use of the test in the general population, if the aim is to rule out the presence of SARS CoV-2 antibodies (Table 1).

### Population screening

Population screening during a pandemic phase requires a highly specific assay, to assure an acceptable PPV in populations with a low sero-prevalence^3^, and additionally a reasonable sensitivity (Table 1). This condition was met for the Wantai ELISAs and Euroimmun IgG ELISA. The Euroimmun IgA and Liaison  CLIA analyzer performed less well, with specificities <95% when testing serum samples from persons exposed to a range of viruses (Table 1)^8^. This led to very low PPV’s of respectively 39% and 26% for the Euroimmun IgA and the CLIA analyzer in low prevalence settings, while the Wantai total Ig continued to perform reasonably well. Tested specimen were obtained from patients with mild, moderate, and severe disease. All patients had detectable antibodies by PRNT50 from day 18 (supplementary data). The severity of disease did not affect the range of detected neutralizing titers or sensitivity of selected assays (Table 1, supplementary data). Due to limitations in sample volumes of mild patients, these were not equally tested in all assays. In addition, future studies are recommended to address the performance of alternative high throughput assays like the Roche Elecsys Anti-SARS-CoV-2 or Abbott SARS-CoV-2 IgG, in correlation to neutralization.

For the RDTs the specificities varied from 85 to 99% (Table 1), although  the overall performance of the Cellex assay with 99% specificity was hampered by its low sensitivity of 80%. Generally, the use of the selected RDT is not recommended in population screening where estimated seroprevalence is mostly <5% and the PPV will be too low for a reliable interpretation (Table 1). A final question in population screening is whether the antibody measurements correlate with functional antibodies that can protect a population during a subsequent exposure. In our analyses, samples testing positive in the Wantai Ig ELISA with an OD ratio > 10 all had detectable levels of neutralizing antibodies which suggests that—using a cut-off- in this assay could be used to indicate presence of neutralizing antibodies. The exact kinetics and functionality of these antibodies in offering protection remains to be determined.

In conclusion, our presented data support decision making for the use of serology in either individual patient care or population-level serological testing. We conclude that for the aim of detecting protective antibodies, the RBD based Wantai ELISA had the best overall performance including the potential to set a cut-off indicating the presence of protective antibodies. The global performance of the selected RDTs is not robust enough for over the counter personalized testing in the population.

## Methods

### Blood samples

All specimen used in the study have been collected and delivered to our diagnostic laboratory for patient diagnostics, and not following a predefined COVID-19 research protocol. To determine specificity of the assays, we used a well-defined panel of 147 serum and plasma samples from 147 individuals exposed to human coronaviruses (HCoV-229E, NL63 or OC43), SARS, MERS), or with a range of other respiratory viruses (adenovirus, human metapneumovirus, influenza A/B, RSV A/B, rhinovirus, Bocavirus, parainfluenzavirus 1 and 3, enterovirus). Specimen from patients with recent cytomegalovirus (CMV), Epstein Barr virus (EBV) or M. pneumoniae infection were included as these have a high likelihood of causing cross reactivity. Sera were collected from 2–3 weeks upon the respiratory infection, and during the acute phase of CMV or EBV. The variation in the number of samples tested per assay was caused by limited sample volume and by the limited  availability of RDTs during the study period.

Sensitivity was calculated by using a total of 187 sera from 107 individuals in the Netherlands, in whom COVID-19 was confirmed by RT-PCR and antibodies were detected by PRNT50. Disease severity varied from (1) Mild, non-hospitalized, (2) Moderate, hospitalized, and (3) Severe, admitted to the intensive care unit. Specimen were taken at different time-points post-onset of disease (supplementary data, Fig. 1). All specimen were stored at −20 °C until use. The variation in the number of samples tested per assay is caused by the fact that validation was part of the acute diagnostic response during the first phase of the COVID-19 pandemic. Initially, a set of 75 patient sera was tested in all assays. These sera were mostly collected from hospitalized patients and involved only three sera from mild patients. To assess the performance of the assays in population screening it is important to involve sera from mild patients so we increased the number of tested sera in the Wantai ELISAs, Liaison, and RDTs. The selection of these assays was based on the best overall performance of Wantai ELISAs in the first analyses and the likely application of Liaison and RDTs in population screening. The exact number of specimen tested per assay varied due to availability of serum. Figure 1 depicts the outcome of the assays per time interval, analyses in mild patients are shown in Table 1, source data can be found in the Supplementary Data 1.

### Ethics declarations

The use of specimen was approved by the Erasmus MC medical ethical committee (MEC approval: 2014–414), which allows the use of clinical data and left-over material from the specimen delivered to our laboratory for diagnostics, unless patients have declared they opted out of this scheme. In addition, the Erasmus MC institutional research committee regulated that all COVID-19 patients admitted to Erasmus MC are asked for permission to use their clinical data and left-over patient material for COVID-19 research purposes. All patients who refused have been excluded from the analyses.

### ELISA

Four selected ELISAs were performed according to manufacturer’s protocol: (1) Wantai SARS-CoV-2 total Ig and IgM ELISAs from Beijing Wantai Biological Pharmacy Enterprise Co., Ltd., China. The ELISAs are coated with RBD antigen. (2) Euroimmun Anti-SARS-CoV-2 IgG and IgA ELISA assays from EUROIMMUN Medizinische Labordiagnostika AG, Lübeck, Germany. The Euroimmun ELISAs are  coated with S1 antigen.

### DiaSorin Liaison XL

The Liaison XL by DiaSorin (Saluggia, Italy), is a semi-automated system using chemiluminescent immunoassay (CLIA) technology for detection of Ab in human samples. The assay is based on S1 and S2 coating antigens. The assays were performed following manufacturer’s protocol.

### Rapid antibody test

The rapid tests we evaluated are (1) Rapid SARS -CoV-2 Antibody (IgM/IgG) Test from InTec utilizing the nucleocapsid protein as antigen (Test lots S2020021505 and GJ20030288), (2) the qSARS-CoV-2 IgG/IgM Cassette Rapid Test (GICA) from Cellex Inc. utilizing both the spike and the nucleocapsid protein (Test lot 20200416WI5513C) and (3) the COVID-19 IgG/IgM Rapid Test Cassette (Whole Blood/Serum/Plasma) from Orient Gene / Healgen (Test lot 2003309), utilizing both the spike and the nucleocapsid protein. All three tests are based on immunochromatography for detection of IgG and IgM specific to SARS CoV-2 in human whole blood (venous and fingerstick) serum or plasma. We performed the tests following the manufacturers’ instructions. Each sample was tested by one test and readout (positive/negative) interpreted by two operators in parallel.

### PRNT 50

An in-house plaque-reduction neutralization test (PRNT50) was used as a reference for this study, because virus neutralization assays are the gold standard in coronavirus  serology. We tested serum and plasma samples for their neutralizing capacity against SARS-CoV-2 (German isolate; GISAID ID EPI_ISL 406862; European Virus Archive Global # 026V-03883) by PRNT50 as previously described by Okba et al.^3^.

### Statistical analysis

The outcome of commercial testing was correlated to functional antibody measurements, to assess likelihood of predicting protective antibody responses. The results of the different ELISAs and RDTs were compared with those detected by PRNT50. For sensitivity calculations only the PRNT50 positive samples were used for the calculations. Specificity was calculated by using the cross reactive panel of non-SARS CoV-2 sera. Graphs were made by using GraphPad Prism version 8 (https://www.graphpad.com). The predictive values were calculated for three scenarios 4% seroprevalence in a general population, 50% seroprevalence in a high-risk sub-population and 95% seroprevalence in confirmed or highly suspect COVD-19 hospitalized patients.

### Reporting summary

Further information on research design is available in the Nature Research Reporting Summary linked to this article.

## Supplementary information

Peer Review File

Reporting Summary

Description of Additional Supplementary Files

Supplementary Data 1

## Data Availability

Source data are provided with this paper. Other data are available from the corresponding author upon reasonable requests. Source data are provided with this paper.

## Source data

Source Data
