# Supplementary material for: An evaluation of COVID-19 serological assays informs future diagnostics and exposure assessment
Source: Nat Commun. 2020 Jul 6;11:3436. doi: 10.1038/s41467-020-17317-y (PMC7338506; doi:10.1038/s41467-020-17317-y)
Supplement: Supplementary file 3 — Description of Additional Supplementary Files [file 41467_2020_17317_MOESM3_ESM.pdf]

Description of Additional Supplementary Files

File Name: Supplementary Data 1
Description: Rough data of the serological laboratory assays are depicted as OD ratio (Wantai and Euroimmun), Arbitrary Units/ml (Liaison) and PRNT titer (PRNT50). The rapid tests are depicted as positive or negative.
