# Supplementary material for: An evaluation of COVID-19 serological assays informs future diagnostics and exposure assessment
Source: Nat Commun. 2020 Jul 6;11:3436. doi: 10.1038/s41467-020-17317-y (PMC7338506; doi:10.1038/s41467-020-17317-y)
Supplement: Supplementary file 1 — Peer Review File [file 41467_2020_17317_MOESM1_ESM.pdf]

Editorial Note:

This manuscript has been previously reviewed at another journal that is not operating a transparent peer review scheme. The manuscript was considered suitable for publication without further review at *Nature Communications*.
